# Predator Diversity Effects in an Exotic Freshwater Food Web

**DOI:** 10.1371/journal.pone.0072599

**Published:** 2013-08-21

**Authors:** Rahmat Naddafi, Lars G. Rudstam

**Affiliations:** 1 Department of Wildlife, Fish and Environmental Studies, Swedish University of Agricultural Sciences, Umeå, Sweden; 2 Cornell Biological Field Station, Department of Natural Resources, Cornell University, Bridgeport, New York, United States of America; Leibniz Center for Tropical Marine Ecology, Germany

## Abstract

Cascading trophic interactions are often defined as the indirect effects of a predator on primary producers through the effect of the predator on herbivores. These effects can be both direct through removal of herbivores [density-mediated indirect interactions (DMIIs)] or indirect through changes in the behavior of the herbivores [trait-mediated indirect interactions (TMIIs)]. How the relative importance of these two indirect interactions varies with predator diversity remains poorly understood. We tested the effect of predator diversity on both TMIIs and DMIIs on phytoplankton using two competitive invasive dreissenid mussel species (zebra mussel and quagga mussel) as the herbivores and combinations of one, two or all three species of the predators pumpkinseed sunfish, round goby, and rusty crayfish. Predators had either direct access to mussels and induced both TMII and DMII, or no direct access and induced only TMII through the presence of risk cues. In both sets of treatments, the predators induced a trophic cascade which resulted in more phytoplankton remaining with predators present than with only mussels present. The trophic cascade was weaker in three-predator and two-predator treatments than in one-predator treatments when predators had direct access to dreissenids (DMIIs and TMIIs). Crayfish had higher cascading effects on phytoplankton than both pumpkinseed and round goby. Increased predator diversity decreased the strength of DMIIs but had no effect on the strength of TMIIs. The strength of TMIIs was higher with zebra than quagga mussels. Our study suggests that inter-specific interference among predators in multi-species treatments weakens the consumptive cascading effects of predation on lower trophic levels whereas the importance of predator diversity on trait mediated effects depends on predator identity.

## Introduction

Indirect effects of predators on basal resources, i.e. top down trophic cascades, are a major driver of the dynamics of ecological communities in both terrestrial and aquatic systems [1], [2]. According to classic theory, trophic cascades emerge through changes in density of species occupying the intermediate trophic levels [density-mediated indirect interactions (DMIIs)]. However there is a growing consensus that nonlethal effects of predators on prey foraging behavior [trait- mediated indirect interactions (TMIIs)] can also drive trophic cascades [2]–[5]. Trait- and density-mediated indirect effects may be independent because these interactions could occur through different pathways in a food web [6]. Ecologists increasingly appreciate the importance of both the TMIIs and DMIIs in the context of predator prey dynamics and trophic cascades [3], [6]–[12].

Under anthropogenic pressure, top trophic levels are typically more susceptible to extinction than their prey resources leading to declines in predator diversity. However, the outcomes of changing predator diversity on indirect interactions are often overlooked [13]. As there is a trade-off between prey foraging success and risk of predation [14], [15] and the non-lethal effect of multiple predators on species interactions can be different from the effect of a single predator [16], [17], the diversity of predators may affect the strength of anti-predator behavioural responses and hence the strength of trait-mediated indirect interactions in both aquatic and terrestrial systems [18]–[21]. Predator diversity may also increase the magnitude of DMIIs [18], [22]. However, intra-guild predation among predators may counteract this expectation [23]; Borer et al. (2005) found no relationship between species diversity and trophic cascade strength [24]. These contradictory predictions suggest a gap in our understanding of the resulting indirect effects of predator diversity. Although resource levels, predator identity, predator diet breadth, abundance and diversity of available prey as well as habitat determine the relative importance of DMII and TMII [2], [9], [25]; it is less clear how it is affected by predator diversity. Further, it is likely that the diversity of functional characteristics of organisms present in the ecosystem rather than the diversity of species per se affect ecosystem function [20], [26]. In this study, we evaluated how the non-lethal and lethal effects of predators with different hunting modes influence the importance of density- and trait-mediated trophic cascades in a freshwater food web.

We used a freshwater three trophic level community consisting of a combination of three predator species with different hunting modes (round goby *Neogobius melanostomus*, rusty crayfish *Orconectes rusticus*, and pumpkinseed sunfish *Lepomis gibbosus*), two invasive filter-feeders (the zebra mussel *Dreissena polymorpha* and quagga mussel *D. rostriformis bugensis*) and a primary producer (phytoplankton). The zebra mussel and quagga mussel, native to the Black and Caspian Sea basin, are successful invasive species in North American and European lakes [27]–[30]. Pumpkinseed and rusty crayfish are native to North America whereas round goby is an invasive species from the Ponto-Caspian region [29]. All three predators are known to consume dreissenids in lakes [29]. Crayfish often wait for prey at a fixed location and respond to olfactory or tactile cues to seek and attack prey [2]. Pumpkinseeds are visually oriented active predators that use suction feeding to dislodge mussels and break the shells with their pharyngeal teeth. Goby feed nocturnally by exploring bottom habitat and probably detect mussels through tactile senses. They also detect moving prey using lateral lines while stationary [31]. We demonstrated that increased predator diversity decrease the strength of trophic cascades only when predators could kill prey but not when the cascading effect was through trait mediated indirect interactions, thus increased predator diversity decreased the importance of DMIIs but not TMIIs.

## Methods

All field collection and laboratory procedures were conducted under the oversight and approval of Cornell University's Institutional Animal Care and Use Committee (Protocol 2006–0088). New York State Department of Environmental Protection as well as Scientific Collecting Permit to Cornell Biological allowed us to sample fish from our sampling sites. The study was not carried out on private land. No specific permissions were required for the sampling locations/activities. Field studies did not involve endangered or protected species. We used traps to catch crayfish and gobies and electrofishing to collect pumpkinseeds. Only round goby was sacrificed after the study. The guidelines of Cornell University's Animal Care and Use Committee were followed for the care of all experimental fish. At the end of experiment, we returned the predators to the sites where they were collected, except for round gobies which were euthanized with an overdose of MS-222.

Oneida Lake is a 207 km^2^ lake with a mean depth of 6.8 m in New York State, USA (43°12' N, 75°54' W). The lake has had abundant zebra mussel populations since 1992. Quagga mussels arrived in Oneida Lake around 2005 [32]. Oneida Lake was once classified as eutrophic, but following water quality improvement efforts and establishment of zebra mussels, the lake appears to have stabilized at a lower productivity level typical of a mesotrophic lake: mean annual total phosphorus (TP) and chlorophyll-a (Chl-a) concentrations changed from 45.9 µg. L^−1^ and 9.2 µg. L^−1^ in 1975 to 22.3 µg. L^−1^ and 3.8 µg. L^−1^ in 2008 [33].

To investigate both lethal (DMIIs) and nonlethal (TMIIs) cascading effects of predators, we conducted a 2×6 factorial designed experiment (replicated three times) using two levels of herbivore treatment (herbivore absent, herbivore present) and six levels of predator treatment [no predator (controls), three individual predators of goby, pumpkinseed, or crayfish (representing single predator species treatment), a combination of 3 individuals of two of the species (2 crayfish and 1 pumpkinseed or 2 crayfish and 1 goby or 2 goby and 1 pumpkinseed) or one individual of all three predator (n = 36). The predator treatments were run two ways: (1) with the predators having access to the mussels (lethal experiments) and thus having the possibility of inducing both DMII and TMIIs on phytoplankton and (2) with the mussels shielded from the predators (non-lethal experiments) allowing only TMII on phytoplankton. These two types of treatments are described in detail below. We collected all animals from Oneida Lake except gobids which were obtained from nearby Lake Ontario. The experiment was conducted separately for zebra and quagga mussels.

To acclimate the mussels to laboratory conditions, the mussels (shell length 7–13 mm) were placed in ten 50-L aerated containers (150 zebra mussels and 150 quagga mussels per container) with natural lake water at ambient temperature in the laboratory for two weeks. The water in the containers was exchanged with new lake water once a day and also 2 h before the experiment began [34]. All predators were kept in separate flow-through aerated round tanks (800 L) containing dechlorinated municipal water originating from Lake Ontario and held in these tanks for three months prior to the start of the experiment. During the acclimation period, we fed the predators small dreissenids (5–13 mm) once a day and then siphoned all crushed shell fragments and feces from the tanks. The siphoned water (approximately 1/8 of the tank) was immediately replaced with dechlorinated water. Two days before the start of the grazing experiment, we kept all experimental predators and mussels (confined to the cages) in the same type of 40-L aquaria (55L×40W×30H cm) that were used in the experiment (see below). This provides the experimental animals a chance to acclimate to each other. The grazing experiments were done in early October 2009 when water temperature (16.5 °C) and initial concentration of Chl a as well as phytoplankton composition was suitable for mussel feeding activity. Phytoplankton composition was dominated by diatoms in Oneida Lake at that time, in particular the 40 µm *Stephanodiscus niagarae* (92% of biovolume). Previous grazing experiments revealed that zebra mussels prefer high quality phytoplankton like diatoms [35] as well as phytoplankton/particles with a size of 7–50 µm [36], 15–45 µm [37], and 30–100 µm [38].

Two sets of experiments were run on the same day with an identical factorial design. In the first set, the mussels were confined to cages (15×15×15 cm) to prevent direct mortality and any direct effects of predators actually touching the mussels. Thus, only chemical risk cues were present that could induce TMII on phytoplankton. In the second set, the predators had direct access to the mussels and could and did feed on them. To initiate the experiments, Oneida Lake water was filtered through a 100 µm mesh net to remove most of the zooplankton, and then transferred to two separate 800-L containers [34]. Water from the two containers was homogenized by exchanging water between the tanks several times. After homogenization, 32 L of this water was poured into each of thirty-six 40-L aquaria. For the herbivore present treatment, each container received 30 zebra mussels. Aeration throughout the experiment mixed the water in such a way that water with risk cues could pass through the screen into the cage housing phytoplankton and herbivores. Flow rate was about 3 L/min. The containers with lake water (phytoplankton) and no herbivores served as controls to correct for changes in phytoplankton biomass due to zooplankton (< 100µm) grazing and/or pigment degradation [34]. Each predator treatment received gobies, crayfish, pumpkinseeds, two paired predators (crayfish-goby, crayfish-pumpkinseed, or goby- pumpkinseed), and all three predators (one individual per predator species). The grazing experiment started once the inhalant and exhalant siphons of the mussels were fully extended and lasted for two hours. Predators were not fed during our 2-h experiment except when they had access to mussels.

Water samples (500 mL) were taken from the centre of the aquaria at the start and end (after 2 h) of the experiment and filtered on GFC filter to determine total phytoplankton biomass (measured by Chl-a content). Chl a concentration was analyzed fluorimetrically after the extraction in 10 mL of 90% buffered acetone (90% acetone, 10% deionized water, 2 drops NaOH per liter) [39]. Initial Chl a concentrations varied within a narrow range in the different treatments (2.16 µg. L^−1^ to 2.36 µg. L^−1^ for the zebra mussel experiments and 1.99 µg. L^−1^ to 2.29 µg. L^−1^ for the quagga mussel experiments). We estimated the proportion of phytoplankton biomass that remained at the end of the experiment ((initial value - consumed value)/initial value) ×100) for each replicate to account for small differences in initial Chl a concentrations among aquaria. All analyses, including calculations of TMII and DMII described below were conducted on the proportion of phytoplankton remaining at the end of the experiment.

TMIIs and DMIIs were estimated according to Wojdak & Luttbeg (2005) [25]:

TMII =  (resources with caged herbivores/average resources with no predator) – 1

DMII =  (resources with deadly predator/average resources with caged herbivores) – 1

The numerator is the amount of resources (proportion of initial phytoplankton biomass) remaining at the end of the trial for a single replicate and the denominator is the average amount of resources remaining for controls with no predators [25]. Subtracting 1 in these equations makes the TMII and DMII measures to equal 0 when there is no indirect interaction. Because we included a no-mussel treatment to measure how phytoplankton biomass were influenced by factors other than mussel feeding, all values were first corrected for changes in phytoplankton biomass in "no mussel" treatment and then used in the TMII and DMIIs equations. All calculations were made using the proportion of phytoplankton biomass that remained in each chamber at the end of the experiment.

Both experiments were performed using the same predators and the same number of quagga mussels as the herbivores the next day.

We used one-way analysis of variance (ANOVA) to compare initial Chl a concentration among predator treatments separately for zebra and quagga mussels. We analyzed predator effects and mussel species effects on the proportion of phytoplankton biomass remaining with a two-way ANOVA that considered predator treatment and mussel species (zebra mussel, quagga mussel) as fixed effects. This analysis was performed separately for lethal and nonlethal trials. We used two-way ANOVA to analyze the effect of mussel species and predator treatment on number of mussels consumed by predators in lethal trails. We performed three-way ANOVA to test the effects of mussel species, type of indirect interaction (TMIIs and DMIIs), and predator treatment on the magnitude of indirect interactions. Predator treatment was treated in different ways in the two-way and three-way ANOVAs. First, we used it to compare no predator, single predator species (goby, pumpkinseed, and crayfish treatments combined), mixture of two predator species, and three predator species treatments to address the effect of predator diversity. We then used it to compare goby, pumpkinseed, and crayfish treatments in single-predator species treatments. All analyses were followed by a Tukey’s test for multiple comparisons and for assessing the effects of predator identity and diversity on prey resources (phytoplankton biomass). Homogeneity of variances was tested with Levene’s test.

## Results

**Figure 1:**
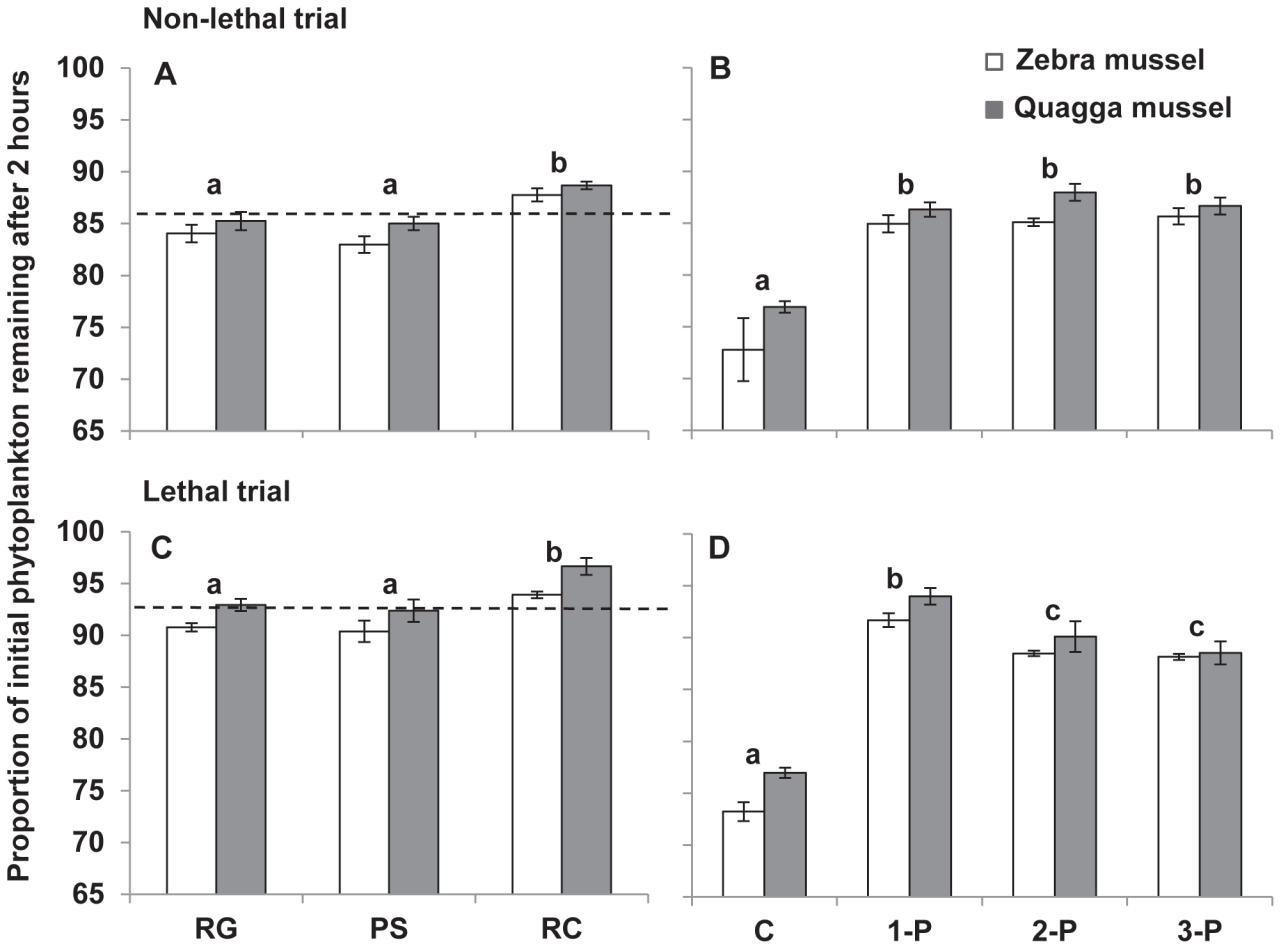
Non-lethal and lethal effects of predators on phytoplankton biomass. Proportions of initial phytoplankton remaining after 2 hours of dreissenids feeding are given in left panels for the single predator species treatments (A, C) and in right panels for the absence and presence of predators along a gradient of predator diversity (B, D) in non-lethal (upper panels) and lethal (lower panels) trials. Values are mean ± 1SE (n = 9 for 1-P and n = 3 for other treatments). RG, round goby treatment; PS, pumpkinseed sunfish treatment; RC, rusty crayfish treatment; C, no predator control treatment; 1-P, single predator species treatment; 2-P, two predator species treatment; 3-P, three predator species treatment. Different single labels (a, b, c) indicate treatments (mussel species combined) that are significantly different (*p*<0.05, Tukey’s test). Dashed horizontal lines (A, C) represent the average effect of single predator species treatment on phytoplankton biomass.

Levene’s test of homogeneity of variances showed that our data were homogenous (*p*>0.05). There was a significant overall effect of treatment type on the initial Chl-a values for both zebra (one-way ANOVA, F_5,66_  = 2.5, *p*<0.05) and quagga (F_5,66_  = 2.6, *p*<0.05) mussel experiments, but this difference was small and there were no significant pair-wise interactions detected with the Tukey’s test (*p*>0.05). Because we used the initial value measured in each experimental units in our calculations, this small difference in initial values should not affect the results. Both lethal and non-lethal effects of predators on dreissenids had strong indirect effects on the biomass of the primary producer, phytoplankton (Table 1). In both lethal and non-lethal trials, the proportion of phytoplankton remaining varied among predator treatments and mussel species (Table 1, Figure 1). Moreover, crayfish had higher indirect effect on phytoplankton than goby and pumpkinseed (Tukey's test, *p*<0.001, Figure 1A,C). In non-lethal trials, there were no differences between the experiments with one predator species and the experiments with combinations of two of the predators or all three predators (Tukey's test, all *p*>0.9, Figure 1B). In lethal trials, one-predator species treatment had the highest indirect effect on phytoplankton among predator treatments (Tukey's test, all *p*<0.01, Figure 1D).

**Table 1:** Results of two-way ANOVAs testing the effect of mussel species and predator treatment on proportion of phytoplankton biomass remaining at the end of experiment in lethal and non-lethal trials among predator diversity (no predator control, single predator species, mixture of two predator, three-predator species) and among single predator species (round goby, pumpkinseed, rusty crayfish) treatments.

			Non-lethal			Lethal		
Source		*df*	Mean square	F	*p*	Mean square	F	*p*
Predator diversity treatment	Mussel species	1	40.0	7.3	< 0.05	29.2	7.9	< 0.01
	Predator treatment	3	205.2	38.0	< 0.001	473.1	130.0	< 0.001
	Mussel species × Predator treatment	3	3.7	0.7	0.6	3.0	0.8	0.5
	Error	28	5.4			3.7		
Single predator species treatment	Mussel species	1	8.5	5.5	< 0.05	23.9	13.7	<0.01
	Predator treatment	2	31.0	19.9	< 0.001	27.2	15.6	< 0.001
	Mussel species × Predator treatment	2	0.5	0.3	0.7	0.2	0.1	0.9
	Error	12	1.6			1.7		

**Figure 2:**
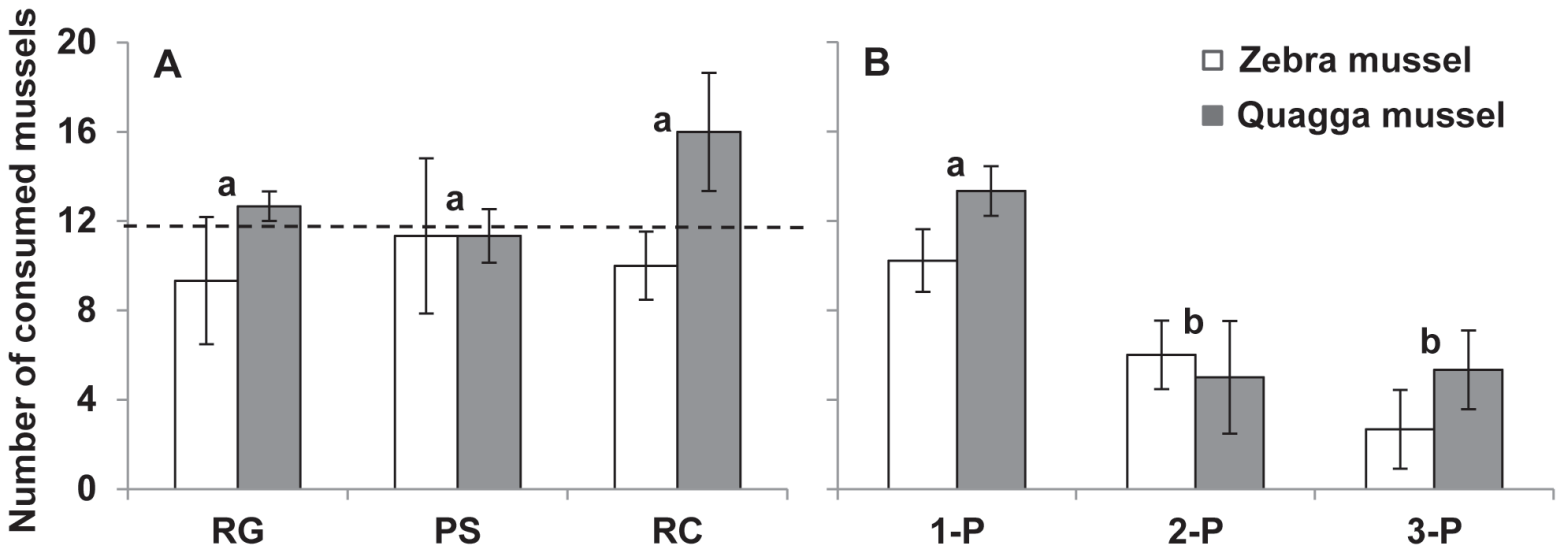
Consumptive effects of predators on mussels in lethal trail. Numbers of consumed mussels are given in left panels for the single predator species treatment (A) and in right panels for a gradient of predator diversity (B). For abbreviations refer to Figure 1. Values are mean ± 1SE (n = 9 for 1-P and n = 3 for other treatments). Different single labels (a, b) indicate treatments (mussel species combined) that are significantly different (*p*<0.05, Tukey’s test). Dashed horizontal line (A) represents the average number of consumed mussels in single predator species treatment.

Mussel species (two-way ANOVA, F_1,12_ = 2.8, *p* = 0.1) and predator species (F_2,12_ = 0.4, *p* = 0.7) had no effect on the number of consumed mussels among single predator species treatment (Figure 2A). When two or three species of predators were present, the number of consumed mussels varied among predator treatments (two-way ANOVA, F_2,12_ = 13.6, *p*<0.001) but not among mussel species (F_1,12_ = 1.1, *p* = 0.3) and was highest in one predator species treatments (Tukey's test, all *p*<0.01, Figure 2B).

**Figure 3:**
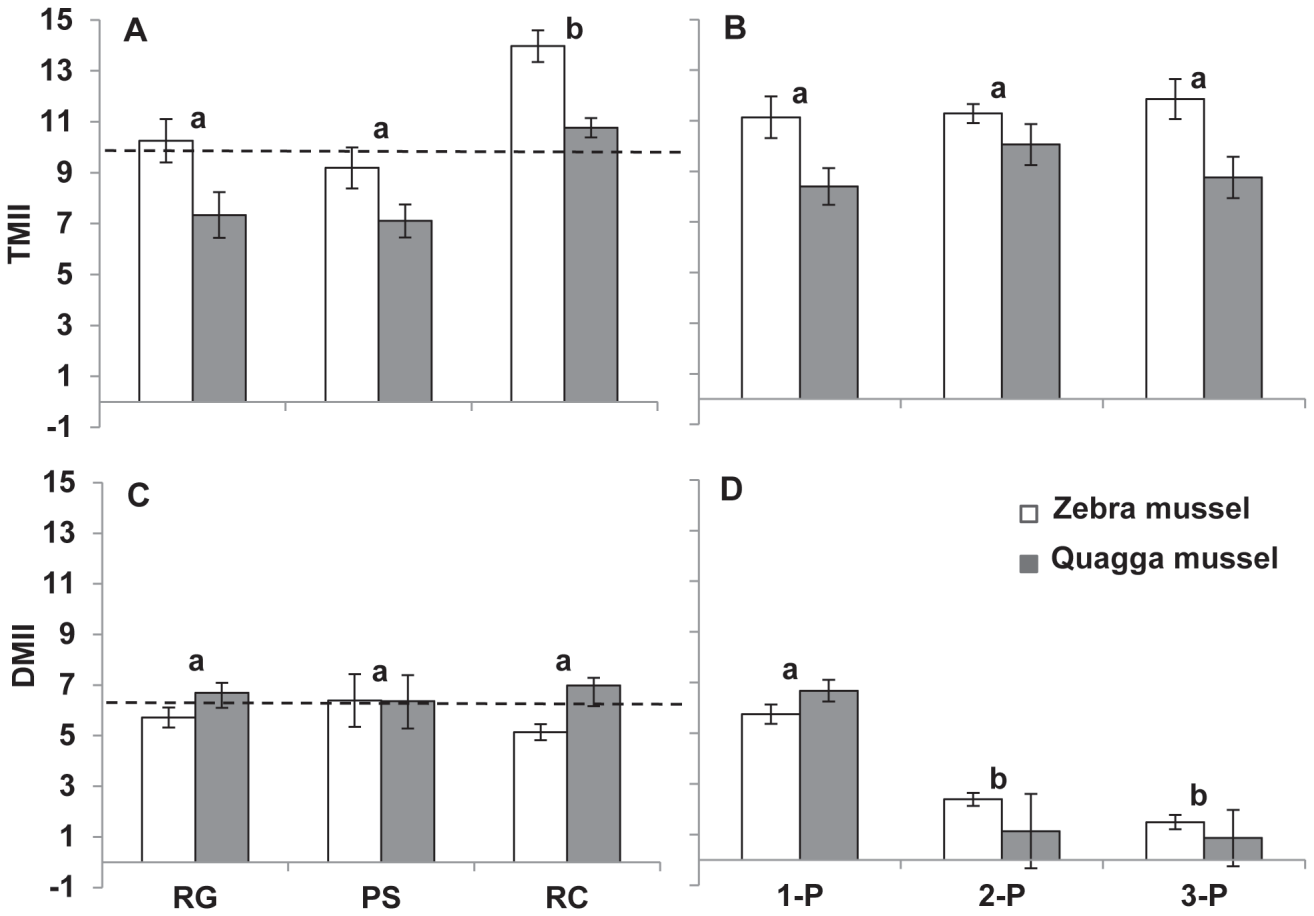
The effect of predators on the strength of indirect interactions. The magnitude of indirect effects of predators on phytoplankton biomass are given in left panels for the single predator species treatment (A, C) and in right panels for a gradient of predator diversity (B, D) on phytoplankton biomass. TMII, trait-mediated indirect interactions (upper panels); DMII, density mediated indirect interactions (lower panels). For abbreviations refer to Figure 1. Values are mean ± 1SE (n = 9 for 1-P and n = 3 for other treatments). Different single labels (a, b) indicate treatments (mussel species combined) that are significantly different (*p*<0.05, Tukey’s test). Dashed horizontal lines (A, C) represent the average magnitude of indirect effects in single predator species treatment.

The strength of indirect interactions varied among predator treatments and mussel species (Table 2). The magnitude of TMII was higher than DMII among predator treatments (*p*<0.01, Table 2, Figure 3). The relative importance of TMII and DMII was dependent on mussel species, as indicated by strong interaction between mussel species × kind of indirect interaction (Table 2): the magnitude of TMII was higher with zebra than with quagga mussels, whereas mussel species had no effect on the magnitude of DMII (Fig 2). The strength of indirect interactions was higher in the crayfish treatment than in the goby and pumpkinseed treatments (Tukey test, *p*<0.01). The magnitude of TMII was higher in the crayfish treatment than in the round goby and pumpkinseed treatments (Figure 3A) but the magnitude of DMII was not different among single predator species treatment (Figure 3C), as indicated by significant interaction between predator treatment × kind of indirect interaction (Table 2). The strength of indirect interactions was greater in the single-predator species treatment than in the other predator treatments (Tukey’s test, *p*<0.01, Figure 3B,D). The strong interaction between predator treatment × kind of indirect interaction, revealed that the effects of risk cues on the strength of indirect interactions was dependent on the kind of indirect interaction (Table 2): the magnitude of TMII in mixture of two predator species and the three predator treatments did not differ from the treatments with single predator species (Figure 3B), but the magnitude of DMII was reduced with increasing predator diversity (Figure 3D).

**Table 2:** Results of three-way ANOVAs testing the effect of mussel species, kind of indirect interaction (TMIIs and DMIIs), and predator treatment on the effect sizes (magnitude) of indirect interactions among predator diversity (no predator control, single predator species, mixture of two predator, three-predator species) and among single predator species (round goby, pumpkinseed, rusty crayfish) treatments.

Source		*df*	Mean square	F	*p*
Predator diversity treatment	Indirect interaction	1	600.2	206.5	< 0.001
	Mussel species	1	20.7	7.1	< 0.05
	Predator treatment	2	29.8	10.2	< 0.001
	Mussel species × Indirect interaction	1	12.0	4.1	< 0.05
	Predator treatment × Indirect interaction	2	53.8	18.5	< 0.001
	Mussel species × Predator treatment	2	1.1	0.4	0.7
	Mussel species × Predator treatment × Indirect interaction	2	3.8	1.3	0.3
	Error	48	2.9		
Single predator species treatment	Indirect interaction	1	114.0	69.1	< 0.001
	Mussel species	1	7.4	4.5	< 0.05
	Predator treatment	2	13.6	8.3	< 0.01
	Mussel species × Indirect interaction	1	30.1	18.3	< 0.001
	Predator treatment × Indirect interaction	2	17.6	10.7	< 0.001
	Mussel species × Predator treatment	2	0.1	0.1	0.9
	Mussel species × Predator treatment × Indirect interaction	2	1.7	1.0	0.4
	Error	24	1.7		

## Discussion

Our study suggests that predator effects on both herbivore density and herbivore feeding rate affect the abundance of primary producers. In our experiments, more phytoplankton remained when dreissenids were subjected to predators due to the suppression of both abundance and feeding rates of the dreissenids (Figure 1, see also [4]). This indicates a strong trophic cascade in this new food web, which is especially important because dreissenids are invasive ecosystem engineers that filter a large volume of water in a relatively short period of time and affect food web dynamics, biodiversity, and function of invaded ecosystems [4], [27], [36], [40]. Moreover, the non-lethal effects of predators are immediate and can influence an entire prey population [3], [7]. In this study the magnitude of TMII was higher with zebra than with quagga mussels. As the cost of TMII (lower feeding rate) should be offset by decreased mortality through predation, we would expect zebra mussels to have lower predation mortality than quagga mussels. This was also the case in a number of our experiments (R. Naddafi & L. Rudstam, unpublished data) and in our measurements of DMII reported here. Although it is possible that zebra mussels were initially dominant over quagga mussels, a more likely explanation is the faster colonization rate of zebra mussels into new environments [41]. On the other hand, lower magnitude of TMII in quagga mussels may allow this species to grow faster and therefore out-compete zebra mussels for food and space. This may in turn result in higher performance of quagga mussels in natural systems if predation levels are low (see [5], [42]). Thus, interspecific differences in TMIIs may affect the competitive interaction between two prey species that use shared resources [42].

The proportion of phytoplankton biomass remaining in single predator species treatment was similar to that in two-predator and three-predator species treatments in non-lethal trials (see Figure 1B) resulting in a lack of predator diversity effect on the strength of TMII. Similarly, Freeman et al. (2009) did not detect any predator specific response in blue mussels when exposed to odors from pairwise combinations of three predators with different attack strategies [43]. However, in some ecosystems, prey species do integrate multiple cues about predators to optimize induced defenses [44]. Therefore, the degree that predator diversity affects TMII and trophic cascades likely is specific to the prey and predator species tested.

The TMII were stronger in the presence of crayfish than in the presence of risk cues from both pumpkinseed and goby (Figure 3A). This is consistent with a generally higher degree of dreissenids morphological responses to crayfish than to other predators (e.g. shell thickness [45]; R. Naddafi & L. Rudstam, unpublished data). Greater shell thickness is typically associated with a reduction in growth rate in mollusks and can result from lower feeding rate [46]–[48]. Decreased filtering rates may be a better response to crayfish than to the two fish species as crayfish are probably more attracted by olfactory stimuli released from live dreissenids feeding on phytoplankton than are fish predators [49], [50]. Prey like dreissenids may be able to balance the magnitude of anti-predator responses with a perceived level of risk to minimize the cost associated with predator avoidance [51]–[53].

In Steffan & Synder’s (2010) study, diverse predator assemblages induced stronger antipredator behaviors in caterpillar than low diversity predator assemblages, which subsequently resulted in a higher biomass of *Brassica oleracea* plants with diverse predators [21]. Our results partly support Steffan & Synder (2010) [21], as single predator species treatments with pumpkinseeds and gobies resulted in lower TMII than two and three predator species treatments (see Figure 3AB). However, it appears that the crayfish treatment alone have stronger effects than the two or three predator species treatments suggesting that the species of predator involved is more important than predator diversity in the magnitude of TMII. TMIIs exceeded DMIIs under our experimental conditions. Elsewhere, both more sedentary predators like goby and active hunter with narrow habitat domain like pumpkinseed cause TMIIs to be dominant [2]. However, our results are specific to the experimental setup. The relative importance of TMII and DMII should depend on the duration of the experiment and the initial number of mussels relative to the feeding rate of the predators.

Predator diversity decreased the strength of trophic cascades when predators were able to change both density and behavior of prey (both DMIIs and TMIIs, Figure 1). This is consistent with fewer mussels consumed in higher predator diversity treatments (Figure 2). In this case, the different species of predators interfered more with each other than the three individuals of the same species. Predator species may interfere or facilitate each other, reflecting a shift between “risk reduction” and “risk enhancement” effects of multiple predators [18]. Our results indicate the risk reduction was more important than the risk enhancement with this set of predators.

Most prey species coexist with a group of functionally different predators and can respond differently to different predators [16], [48]. It is not surprising that increased predator diversity dampens trophic cascades if predators interfere with each other, possibly even preying on each other (intra-guild predation, [11]). Although we did not observe predation events between our predators, they did interfere with each other and at one occasion, a crayfish even injured a goby. Behavioural interference between different species of predators are often strong [18] and may be more intense than the between members of the same species of predator ([54], [55]; but see [56]).

In this study, only the magnitude of DMIIs decreased with predator diversity, not the magnitude of TMIIs. TMII depends only on the mussel’s reaction to risk cues, and should be less affected by behavioural interference among predators. How the diversity and complexity of food webs affect both trait- and density mediated interactions among species is important for predicting effects of changes in biodiversity on ecosystem function [10].
